# Impact of the COVID-19 Pandemic on Psychiatric Admissions to a Large Swiss Emergency Department: An Observational Study

**DOI:** 10.3390/ijerph18031174

**Published:** 2021-01-28

**Authors:** Julia Ambrosetti, Laura Macheret, Aline Folliet, Alexandre Wullschleger, Andrea Amerio, Andrea Aguglia, Gianluca Serafini, Paco Prada, Stefan Kaiser, Guido Bondolfi, François Sarasin, Alessandra Costanza

**Affiliations:** 1 Emergency Psychiatric Unit, Department of Psychiatry and Department of Emergency (UAUP), Geneva University Hospitals (HUG), 1211 Geneva, Switzerland; laura.macheret@hcuge.ch (L.M.); aline.folliet@hcuge.ch (A.F.); 2 Adult Psychiatry Division, Department of Psychiatry, University Hospital of Geneva (HUG), 1205 Geneva, Switzerland; alexandre.wullschleger@hcuge.ch (A.W.); stefan.kaiser@hcuge.ch (S.K.); 3 Department of Neuroscience, Rehabilitation, Ophthalmology, Genetics, Maternal and Child Health (DINOGMI), Section of Psychiatry, University of Genoa, 16132 Genoa, Italy; andrea.amerio@unige.it (A.A.); andrea.aguglia@unige.it (A.A.); gianluca.serafini@unige.it (G.S.); 4 IRCCS Ospedale Policlinico San Martino, 16132 Genoa, Italy; 5 Department of Psychiatry, Tufts University, Boston, MA 02111, USA; 6 Department of Psychiatry, Faculty of Medicine, University of Geneva (UNIGE), 1206 Geneva, Switzerland; paco.prada@hcuge.ch (P.P.); guido.bondolfi@hcuge.ch (G.B.); francois.sarasin@hcuge.ch (F.S.); alessandra.costanza@unige.ch (A.C.); 7 Service of Liaison Psychiatry and Crisis Intervention (SPLIC), Department of Psychiatry, Geneva University Hospitals (HUG), 1211 Geneva, Switzerland; 8 Emergency Medicine Unit, Department of Emergency, Geneva University Hospitals (HUG), 1211 Geneva, Switzerland; 9 Department of Psychiatry, ASO Santi Antonio e Biagio e Cesare Arrigo Hospital, 15121 Alessandria, Italy

**Keywords:** coronavirus, COVID-19 pandemic, depression, emergency department, public mental health, psychiatric admissions, psychotic episode, substance use disorder, suicide, suicidal behavior

## Abstract

The coronavirus disease 2019 (COVID-19) pandemic is a public health emergency with profound mental health consequences. The psychiatric emergency department (ED) plays a key role during this mental health crisis. This study aimed to investigate differences in admissions at a Swiss psychiatric ED from 1 April to 15 May during a “pandemic-free” period in 2016 and a “during-pandemic” period in 2020. The study included 579 consultations at psychiatric ED in the “during-pandemic” period and 702 in the “pandemic-free” period. Sociodemographic and clinical characteristics were compared, and logistic regression analysis was performed to identify variables associated with psychiatric admissions during the pandemic. A reduction in total psychiatric ED admissions was documented during COVID-19. Logistic regression analysis predicted the independent variable (ED admission during the pandemic) and estimated odds ratio (OR) for being unmarried/not in a relationship, arrival in an ambulance, suicidal behavior, behavioral disorders and psychomotor agitation. Though only statistically significant in bivariate analysis, patients were also more likely to be involuntarily hospitalized. This picture appears to be reversed from a sociodemographic and clinical point of view to our observation of psychiatric ED consultation in 2016. These findings highlight that the reduction in psychiatric ED admissions during the pandemic seems to be associated with living alone and more severe psychopathologies, which must alert psychiatrists to ensure access to mental health care in times of pandemic.

## 1. Introduction

The World Health Organization declared the novel coronavirus disease 2019 (COVID-19) outbreak a Public Health Emergency of International Concern and then a pandemic [1]. The first case of severe acute respiratory syndrome coronavirus 2 (SARS-CoV-2) infection was announced in the canton of Geneva on 26 February [2]. On 13 March, the Federal Council adopted severe public policies to restrict population movement and curb disease spread, including banning groups of more than 100 people, closing schools and universities, and introducing initial border controls with neighboring countries [2]. The situation was then qualified to be “extraordinary” by the Federal Council on 16 March, which banned any public or private events and ordered closures of stores, restaurants, public leisure activity facilities, and entertainment venues, with only grocery stores, post offices, banks, and pharmacies remaining open. The Army and Civil Protection agency were then tasked with assisting the health care system and reinforcing border controls. The population was also asked to stay at home, and, unlike some other European countries, no strict lockdown with stay at home orders was established [2]. These restrictive measures were progressively attenuated from 27 April onwards.

The COVID-19 pandemic is significantly affecting the health, safety, and wellbeing of both individuals and communities [3]. The effects on individuals (e.g., insecurity, confusion, isolation, and stigma) and communities (e.g., school and workplace closures, economic loss, and inadequate or chaotic response to medical necessities) can precipitate a wide range of mental health consequences [3,4,5], including emotional reactions, unhealthy behaviors, and the development of psychiatric conditions in severe cases [6,7]. These consequences have been reported in the general population [8,9,10,11], as well as in vulnerable individuals [12,13], patients with a history of mental health disorders [5,14], and healthcare professionals [10,12,15,16,17]. Moreover, it is likely that these mental health effects will have far-reaching consequences that may even peak after the actual pandemic [18,19]. Psychiatric emergency departments (ED) play a key role during such mental health crises, with collected outcomes providing early insights into the course of a crisis [1,20,21,22].

To the best of our knowledge, only a few studies [23,24,25,26,27,28,29] have investigated quantitative/qualitative differences in psychiatric admissions to psychiatric EDs between the prepandemic period and during the COVID-19 pandemic. Therefore, the present study aimed to compare the sociodemographic and clinical characteristics of patients presenting to the adult psychiatric ED of the University Hospital of Geneva (HUG) in Switzerland between the same time period in two separate years, including a “pandemic-free” period (1 April 2016 to 15 May 2016) [30] and a “during-pandemic” period (1 April 2020 to 15 May 2020).

## 2. Materials and Methods

### 2.1. Participants

The HUG, with its somatic and psychiatric EDs, offers psychiatric emergency care 24 h per day, serving the entire population of almost 500,000 inhabitants in the Geneva canton. This observational, retrospective study recruited 579 admissions to the adult division of the psychiatric ED of the HUG in Switzerland between 1 April 2020 and 15 May 2020. Their number and characteristics were compared to 702 admissions at psychiatric ED between 1 April 2016 and 15 May 2016. We did not establish any inclusion or exclusion criteria: all the patients admitted to ED who required a psychiatric medical evaluation in the considered period were included. Patients consent was waived because we argued that requesting consent would have introduced a selection bias. The research project may be validly considered to be in a higher interest than the interests of the persons concerned when it can be expected to lead to the acquisition of knowledge that will benefit future patients or that could not otherwise be acquired.

This study was conducted in accordance with the Declaration of Helsinki, as revised in 2013 [31]. This study was approved by the Research Ethics Committee of Geneva under the registration number of 2020-01510 (Approval date: 29 June 2020).

### 2.2. Assessment

We collected sociodemographic and clinical data similar to a previous study conducted at the same institution [30]. For each visit, we determined the diagnosis at admission based on the Echelle Suisse de Tri EST^®^ (HUG, Geneva, Switzerland). The EST^®^ is a screening tool recommended by the Swiss Society for Emergency Medicine and Rescue and is currently used in the three language regions of Switzerland, as well as in France and Belgium. We also collected sociodemographic data (sex, age, familial and residential status, and nonmigrant/migrant status), modality of access to the ED (ambulance, police, and self-referral), and the discharge decision made by the ED psychiatrist (nonvoluntary/voluntary hospitalization and returning home).

Finally, the degree of urgency was determined according to the Echelle Suisse du Tri (EST^®^). The EST^®^ scale has four degrees of severity: degree 1 (a very urgent condition, dangerous to life), degree 2 (a pathological situation that is not life-threatening, but which is likely to worsen quickly), degree 3 (a pathological situation where time is not a critical factor, and the state of the patient at arrival is considered stable), and degree 4 (a medical condition considered stable and not requiring emergency care).

### 2.3. Statistical Analysis

Continuous data for sociodemographic and clinical variables were represented as means with standard deviations (SD), while categorical variables were represented as counts with percentages. The Kolmogorov-Smirnov test was performed to test normal distributions of continuous variables.

The total sample was divided into two subgroups based on the HUG ED admission date. Subjects admitted from 1 April 2016 to 15 May 2016 represented the first subgroup [30] (named “pandemic-free”sample), and subjects admitted from 1 April 2020 to 15 May 2020 represented the second subgroup (named “during-pandemic” sample). Pearson’s chi-squared test with the Yates correction or the *t*-test for independent samples was used to compare categorical and continuous variables, respectively, between these subgroups. As the independent variable, we chose ED admission during the pandemic. The variables included in the regression analysis are those significant to the bivariate analysis.

All statistical analyses were performed using the Statistical Package for Social Sciences (Version 25.0, SPSS; SPSS Inc., Chicago, IL, USA) for Windows, and the significance was set at *p* < 0.05 (two-tailed).

## 3. Results

Overall, our findings showed fewer total psychiatric admissions to the ED during the pandemic period than during the “pandemic-free” period with 702 consultations in 2016 vs. 579 consultations in 2020 (decrease of 17.5%).

Patients admitted to the psychiatric ED during the COVID-19 pandemic were more frequently unmarried/not in a relationship and separated/divorced (*p* < 0.001), were less likely to be self-referred, arrived more often by ambulance (*p* < 0.001), were more frequently admitted overnight (*p* = 0.005), had a more severe degree of emergency according to the EST^®^ scale (Degree 1:18.1% vs. 13.7%; *p* = 0.009), and were more likely to be involuntarily hospitalized after their psychiatric consultation in the ED (*p* = 0.032) compared to pandemic-free period. The diagnoses for admissions which increased the most during the 2020 pandemic period were suicidal behavior, behavior disorder (among adults and elderly), and psychomotor agitation (*p* < 0.001). Depression/anxiety was still the most frequent diagnosis for admission, but decreased from 44.2% in 2016 to 30.2% in 2020 (Table 1).

Logistic regression analysis predicted the independent variable (ED admission during the pandemic) and estimated odds ratio (OR) for: unmarried/not in a relationship (*p* < 0.001, OR = 1.754), separated/divorced (*p* < 0.001, OR = 1.938), arrival in an ambulance (*p* < 0.001, OR = 2.407), suicidal behavior (SB) (*p* < 0.001, OR = 2.410), behavioral disorder in adults and the elderly (*p* < 0.001, OR = 2.066), and psychomotor agitation (*p* < 0.001, OR = 4.704) (Table 2).

## 4. Discussion

In this study, we analyzed quantitative and qualitative differences in sociodemographic and clinical characteristics of patients admitted to a Swiss psychiatric ED during the COVID-19 pandemic (1 April 2020 to 15 May 2020) and during the same timeframe during a pandemic-free period (1 April 2016 to 15 May 2016).

In the “pandemic-free” sample analyzed in the 2016 previous study [30], a total of 702 consultations at psychiatric ED were enrolled, with depression and anxiety without urgency or severe features (Degrees 2–4 according the EST^®^ scale) being the most common type of presentation. However, during the pandemic, our patient sample exhibited different characteristics.

From a quantitative perspective, our study documented a reduction in the total number of psychiatric admissions to the ED during the COVID-19 pandemic. This finding is consistent with recently published studies comparing the total number of these types of admissions between 2019 and 2020 for all patient ages [23,24,26,27,29], specifically for ED (Table 3). We hypothesize that these observed declines in urgent consultations for mental health services during the pandemic have resulted from people being told to stay at home and to consider hospitals (in particular, the ED) a dangerous place to visit because of a higher risk of infection.

From a qualitative perspective, a significant increase was noted in the number of consultations to the psychiatric ED from patients who were unmarried/not in a relationship or separated/divorced, which is similar to the findings of a retrospective study performed during the COVID-19 outbreak in an Italian ED [24]. In this study, people who lived alone had an increased frequency of consulting a psychiatric ED, especially during the lockdown period. Moreover, considered together, the clinical characteristics of our “during-pandemic” sample imply that patients who were admitted to the psychiatric ED during the COVID-19 pandemic had more severe psychopathological states, as confirmed by the above-mentioned previous study [24]. In particular, the diagnoses for admissions which increased the most during the 2020 pandemic period were suicidal behavior, behavior disorder (among adults and elderly), and psychomotor agitation.

In contrast to our findings, Aly et al. [25] showed an increase in consultations for nonaffective psychotic disorders and bipolar disorder, which we did not see in our sample. Diagnoses of psychotic episodes actually decreased, and diagnoses of manic/hypomanic episodes remained mostly stable. One hypothesis for this discrepancy is that patients with psychotic disorders or mania/hypomania were more likely to receive a primary diagnosis of psychomotor agitation during the pandemic period, with only a secondary diagnosis of psychosis or bipolar disorder. The same authors reported a lack of change in the total number of psychiatric emergencies; however, their proportion of total ED consults was higher, and their diagnosis was changed. In particular, the risk of suicidal behavior increased among patients admitted to the ED.

An increased suicide risk, which was evident also in our sample and in the literature [24,25], is of particular interest because of its transdiagnostic nature, as well as its possible occurrence in sociodemographic conditions documented during the pandemic period. Suicidality risk has possible fatal implications, but possibilities of prevention exist, if any metabolic, environmental, psychological, or biological trigger mechanisms are recognized [32,33,34,35,36,37]. Only a few studies have investigated how epidemics affect suicidality [13,38,39]. Two studies have reported an increase in suicide deaths during epidemics: one in the United States during the 1918–1919 Spanish Flu epidemic [40] and the other among older people in Hong Kong during the 2003 Severe Acute Respiratory Syndrome (SARS) epidemic [41]. However, multiple several authors have noted that the COVID-19 pandemic and its mitigation policies have led to factors known to precipitate suicide [13,38,42,43], including social isolation/entrapment/loneliness, financial stressors, increased alcohol consumption, increased domestic violence, access to lethal means, intensive exposure to stories of hopelessness (through the media), emerging or exacerbated psychological and psychiatric suffering, barriers to mental and somatic health, and stigma [13,38,42,43]. In addition, case reports of COVID-19-related suicides have begun to appear in the literature [44,45].

Our findings can support some insights into what this means for clinical practice, for example, in terms of organization of EDs, access to mental health care and prevention could be improved.

In terms of organization of EDs, a main resource could be represented by temporarily clearly dividing psychiatric ED entrances from those of somatic EDs (including waiting rooms or triage desks, for example), in order to improve the sense of security of psychiatric patients who fear contagion. Currently, psychiatric EDs are not always equipped with high isolation standards against infectious respiratory diseases: this could be an aspect to address. The media, also, could encourage patients suffering for mental illness to go to the EDs on time as usually they do, without waiting for the worst consequences, by encouraging them that they will find a safe place dedicated to them.

In terms of access to mental health care and prevention, it would be useful to encourage mainly two means: a greater mobility of psychiatrists and psychiatric nurses in going to their homes and the use of telepsychiatry, which appears to be increasingly emerging. Telepsychiatry could promote continuity of care for psychiatric patients at the community level, remotely supporting them to cope with some feelings, which might be exacerbated during health emergencies and associated imposed social distancing measures. Beyond the reluctance that psychiatrists can understandably manifest towards technological devices for fear that they will transform the type of interpersonal relationship or even accentuate the stigma, telepsychiatry can instead be considered a means to remotely support patients to cope with their loneliness, feelings of diminished social connectedness, hopelessness, and helplessness that are, for example, significantly associated with suicidality and might be exacerbated during health emergencies and associated imposed social distancing measures. Telepsychiatry medicine, in this context, has been specifically used in psychiatric EDs and acute settings [46,47,48,49,50,51]. Perhaps, in an unexpected and paradoxical manner, the COVID-19 pandemic could create an opportunity to overcome some normative, technological, and cultural barriers [52].

### Limitations and Strenghts

It is important to note that our study had several limitations. First, data collection for our pandemic sample started after the first wave of the epidemic had already begun; however, we specifically used the time frame between 1 April and 15 May 2020, because it matched the dates used in our previous 2016 [30] study, although the WHO deemed COVID-19 to be a pandemic on 11 March 2020 [1]. Second, we did not analyze differences between specific countries during the interpretation of our data. For example, unlike in Italy, where the study to which we have referred most was conducted [24], the Swiss lockdown had different characteristics, including being substantially more permissive. Third, we investigated only the first wave of COVID-19 contagion. Information about the second wave, which is still ongoing, may also add important insights. Fourth, there may be changes between 2016 and 2020, for example, regarding the organization of health care or the socio-economic context, which we did not analyze. Further, we used 2016 data. More recent data would have made the findings more comparable. Internal statistics of years 2017–2019 globally showed a stable number of consultations in our psychiatric ED, but, because of structural and organizational reasons, these data are not usable in the current state and need further investigation. Finally, further analysis of national registries data that go beyond the sample size of the present study and the previously published studies on psychiatric admissions at EDs during the pandemic is needed.

One strength of this study was that we examined the exact same time frame in 2016 and 2020, with analyses of the same variables, thus eliminating possible biases resulting from seasonality or other factors. In addition, our sample size was also larger than previous studies conducted in psychiatric EDs.

## 5. Conclusions

Our findings highlight that the reduction in psychiatric ED admissions during the pandemic seems to be associated with living alone and more severe psychopathologies, which has to alerting psychiatrists to ensure access to mental health care in times of pandemic. Moreover, the underlying factors contributing to the observed changes in mental health care utilization, particularly its reduction, have not yet been widely studied, and further research is necessary to identify these factors and, thereby, ensure uninterrupted access to mental health services.

## Figures and Tables

**Table 1:** Comparison of sociodemographic and clinical characteristics of patients admitted to the psychiatric emergency department (ED) during a “pandemic-free” period in 2016 and “during pandemic” period in 2020.

Characteristic	“Pandemic-Free” Sample (*n* = 702)	“During-Pandemic” Sample (*n* = 579)	Chi-Squared/ *t*-Test	*p*-Value
Female Gender, *n* (%)	384 (54.7)	300 (51.8)	1.063	0.303
Current age, year, mean ± SD	40.91 ± 17.60	40.58 ± 17.11	−0.337	0.736
Familial status, *n* (%)				
Unmarried/not in relationship	353 (50.3)	335 (57.9)	24.026	<0.001 *
Married/in a relationship	210 (29.9)	109 (18.8)		
Separated/divorced	108 (15.4)	116 (20.0)		
Widowed	31 (4.4)	19 (3.3)		
Residential status, *n* (%)				
Private residence	571 (81.3)	467 (80.6)	10.850	0.013 *
Foster home, hotel	83 (11.8)	71 (12.3)		
Homeless	37 (5.3)	41 (7.1)		
Migrant	11 (1.6)	0 (0.0)		
Referral source, *n* (%)				
Private psychiatrist	13 (1.9)	4 (0.7)	89.724	<0.001 *
General practitioner	30 (4.3)	11 (1.9)		
HUG	78 (11.1)	16 (2.8)		
Self-referral	330 (47.0)	223 (38.5)		
Police	82 (11.7)	60 (10.4)		
CAMSCO	3 (0.4)	2 (0.3)		
By Ambulance	166 (23.6)	263 (45.4)		
Urgency degree, according to EST^®^, *n* (%)				
Degree 1	96 (13.7)	105 (18.1)	11.620	0.009 *
Degree 2	312 (44.4)	268 (46.3)		
Degree 3	265 (37.7)	173 (29.9)		
Degree 4	29 (4.1)	33 (5.7)		
Diagnosis, *n* (%)				
Psychotic episode	77 (11.0)	18 (3.1)	93.838	<0.001 *
Manic/hypomanic episode	13 (1.9)	10 (1.7)		
Depression/anxiety	310 (44.2)	175 (30.2)		
Suicidal behavior	82 (11.7)	122 (21.1)		
Substance use disorder	70 (10.0)	50 (8.6)		
Behavioral disorder (among adults and elderly)	80 (11.4)	112 (19.3)		
Psychomotor agitation	12 (1.7)	38 (6.6)		
Somatic problem	58 (8.3)	54 (9.3)		
Arrival hour, *n* (%)				
8–19	403 (57.4)	282 (48.7)	10.653	0.005 *
19–24	191 (27.2)	178 (30.7)		
24–8	108 (15.4)	119 (20.6)		
Arrival day, *n* (%)				
Weekday	499 (71.1)	431 (74.4)	1.650	0.199
Weekend	203 (28.9)	148 (25.6)		
Type of discharge, *n* (%)				
Voluntary admission	123 (17.5)	90 (15.5)	8.783	0.032 *
Involuntary admission	68 (9.7)	78 (13.5)		
Private residence	389 (55.4)	335 (57.9)		
Others	122 (17.4)	76 (13.1)		
Duration of visit, in hours mean ± SD	7.03 ± 6.65	6.44 ± 6.32	−1.911	0.056

Abbreviations: CAMSCO, Consultation Ambulatoire Mobile de Soins Communautaire; HUG, University Hospital of Geneva, *p* *, statistically significant.

**Table 2:** Logistic regression analysis of the relationships between potential explanatory variables and psychiatric ED admissions in the “during-pandemic” sample.

Characteristic	*p*-Value	OR	95% CI for EXP
Unmarried/not in relationship	<0.001 *	1.754	1.331–2.312
Separated/divorced	<0.001 *	1.938	1.357–2.766
Arrival in ambulance	<0.001 *	2.407	1.873–3.093
Emergency degree 1 based on EST^®^	0.280	0.828	0.588–1.167
Suicidal behavior	<0.001 *	2.410	1.731–3.354
Behavioral disorder (in adults and elderly)	<0.001 *	2.066	1.477–2.890
Psychomotor agitation	<0.001 *	4.704	2.320–9.537
Night admission (arrival hour 24 –8 h)	0.286	1.183	0.869–1.611
Involuntary admission	0.283	1.231	0.843–1.797
Constant	<0.001 *	0.015	

Abbreviations: EST^®^, Echelle Suisse du Tri; *p* *, statistically significant.

**Table 3:** Previous studies comparing ED admission between a control and a during pandemic period.

Authors	Control Sample (*n*)	During Pandemic Sample (*n*)	Country	Decrease in Number in Psychiatric ED Admissions
Capuzzi et al., 2020 [23]	338 (two EDs) (22 February 2019–5 May 2019)	225 (two EDs) (21 February 2020–3 May 2020)	Italy	33%
Montalbani et al., 2020 [24]	133 (1 January 2020–3 May 2020)	58 (11 March 2020–3 May 2020)	Italy	56%
Pignon et al., 2020 [26]	1224 (three EDs) (19 March 2019–15 April 2019)	553 (three EDs) (17 March 2020–13 April 2020)	France	55%
Goncalves et al., 2020 [27]	1633 19 March 2020–2 May 2020)	780 (19 March 2020–2 May 2020)	Portugal	52%
Beghi et al., 2020 [29]	910 (four EDs) (9 March 2020–3 May 2020)	778 (four EDs) (9 March 2020–3 May 2020)	Italy	15%

## Data Availability

The data are available on request.
